# Social-Psychological Satiety: Empirical Study of a New Phenomenon

**DOI:** 10.3390/bs9120138

**Published:** 2019-12-04

**Authors:** Tatiana Drobysheva, Ivan Larionov

**Affiliations:** 1 Laboratory of social and economic psychology, FSFES Institute of Psychology, Russian Academy of Sciences, 13-1 Yaroslavskaya str., Moscow 129366, Russia; 2 FSFEI of HE State Academic University for the Humanities, 26, Maronovskiy per., Moscow 119049, Russia; ivlar16@gmail.com

**Keywords:** social psychology of the city, satiety, the living conditions in the metropolis, forms of satiety, the hedonistic and communicative forms of satiety

## Abstract

This article is devoted to the development of a new phenomenon in the field of social psychology of the city—satiety with living conditions in a metropolis. The study involved 87 people aged 18–30 years. The aim of the study was to identify and analyze the expression of the satiety of young Moscovites with living conditions in the metropolis, and its dependence on the sources of experience of this state. It was shown that satiety as a state of the human psyche performs the function of a protective mechanism from an overabundance of various stimuli from the surrounding world. The satiety with living conditions in the metropolis is a complex state that is caused by a variety of phenomena of social life in the urban environment: Excessive communication, an abundance of information, overpopulation, forced informatization of the urban environment, high rate of life, etc. It was found that satiety with living conditions in a metropolis has different forms of expression, such as emotional experiences and behavioral reactions. The article outlines perspectives for further research on the phenomenon of satiety in living conditions in a megacity in the direction of types, factors, and mechanisms of satiety.

## 1. Introduction

The actuality of research of social-psychological satiety is due to the constant growth of urbanization processes worldwide [1]. According to data of specialists, the rate of relocation from villages and the countryside to big cities has significantly increased over the last six decades. In 2007, for the first time in human history, the number of city dwellers surpassed that of villagers. Data show that the number of citizens is growing much faster than numbers of villagers: From 1950 to 2014, world urban population has increased 5.2 times, while rural population only 1.9 times [2].

The annual growth of the urban population leads to significant changes in various spheres of citizens’ lives. Urban lifestyle and urban culture are increasingly spreading into rural areas of most countries of the world [3]. This process has an impact on the development of the economy, the formation of the population’s way of life, etc. In big cities (especially metropolises), new goods and services are constantly appearing; this increases the flow of information transmitted through advertising (posters, promo actions, videos, etc.). The increase in urban population contributes to the growth rate, rhythm of life, and changes in the nature of communication processes [4,5]. An increasing amount of information includes a variety of incentives for the urban environment, which contributes to the oversaturation of citizens in the living conditions provided by the megacity. Furthermore, research on urban environments, personal space, and architecture show that citizens are forced to change behavioral and psychological reactions in different organizations of personal and public space [6,7,8,9,10].

The phenomena of urban life are the subject of research by specialists in various areas of psychology—social psychology, psycholinguistics, clinical psychology, psychology of perception, etc. [11,12,13,14]. Analysis of the latest Russian psychological research clearly shows interest in studying the psychological effects of living in a megacity [12,15,16,17]. These researches in the field of social psychology of the city are connected with analysis of different characteristics of living conditions in a megacity and their effects on psychological well-being, mental health, etc. The phenomenon of social-psychological satiety with such living conditions can be positioned as one of the main objects for this new research direction. Within the framework of a new socio-psychological approach to the study of this phenomenon, the emphasis should be placed on finding and analyzing its sources in the urban environment. The special interest of the present study focuses on objective and subjective factors of social-psychological satiety with living conditions in megacities and the interrelationship between satiety, value systems, and the urban identity of modern city dwellers.

The satiety manifests itself in all aspects of mental activity, influencing both the very processes of perception—processing and memorizing information about the world—and the social interaction of each individual. The satiety and its manifestations cause negative effects on social cooperation—the lack of empathy makes it hard to establish close relationships, make new friends, create a family, understand new information, etc. Social-psychological satiety as a state of the psyche performs the function of a protective mechanism against an overabundance of various stimuli from the surrounding world. The modern citizen is constantly exposed to the environment, oversaturated with information, and this causes satiety. According to Simmel, this phenomenon leads to several negative effects—impersonal and unemotional attitude to objects and environmental phenomena, loss of interest in life, superficial communication, and a threat to the psychological well-being of the individual.

## 2. Materials and Methods

The purpose of this study is to distinguish and analyze the severity of the state of social-psychological satiety with the living conditions in their connection with sources of this state.

The main hypothesis is that the contents of satiety vary depending on the social phenomena that act as its sources. According to this, various forms of social-psychological satiety can be distinguished.

The sample in this study involved 87 respondents, aged from 19 to 30, 43%—males, 57%—females. For this study, we specially selected respondents of this age because they are leading active lives in an urban environment—they study at universities, build careers, and have active communication in their free time.

The study was performed using the following methods of research—first of all, we used a focus group method to discuss phenomena of social-psychological satiety. The focus group was conducted by I.V. Larionov in the form of a group discussion about the nature of satiety, as well as the sources and manifestations of satiety. A total of 30 2nd year students took part in the 1 h discussion. The discussion was recorded on a voice recorder, then transcribed into text. We took assessments from the focus group with the help of content analysis and used Likert scaling to construct original questionnaires [17]. The first questionnaire included assessments about 8 manifestations of satiety; the second included assessments about 8 sources of this phenomenon. Additionally, we used questionnaires to determine socio-demographic characteristics of respondents, urban identity according to M. Lalli [18], value orientations [19], and associative tests. The results of associative tests were used to determine the structure of social representations about social-psychological satiety, according to the prototypical analysis by P. Verges [20]. We also employed descriptive statistics, frequency and correlation analysis (r-Spearman), for data analysis.

At the first stage, we identified the content and structure of social representation of satiety in the group of respondents. The main purpose of this stage was to reveal content identification interpretations of the phenomenon of satiety in the common mindset (without mentioning its sources) among the young respondents. To make this, we used the prototypical analysis of Verges.

At the second stage of the study, an analysis of the manifestation of satiety with living conditions in a megacity and the analysis of the most significant phenomena of urban life, considered as sources of its particular type, was carried out. Correlation analysis of sources and manifestations of satiety was focused on the allocation of various forms of social-psychological satiety.

## 3. Results

An analysis of associations with the word “satiety” was made using the program SPSS 22.0 (Table 1). To identify the structural status of each element, two indicators were used: The frequency of the association and the rank of its appearance in the answers of respondents. The central elements are high-frequency associations with low ranks, which are in the upper left square. The lower right square contains the periphery of social representations—high-ranked and rarely used associations. The other two squares include elements in the low ranks and high frequency (upper right square), and low frequency and high ranks (lower left square). Many researches agreed that these elements could be considered as sources of potential change.

The analysis of the structure of social representation shows that the central elements are assessments about sufficient amounts of or excess of something—negative or positive emotional experiences. In other words, the central element (core) of social representation contains ambivalent emotional experiences. We can see the same situation in the content of the periphery and the potential sources of change—these zones contain both positive and negative assessments. It is important to mention that the zone of potential change contains assessments about different living conditions in the modern city—crowd, public transport, rhythm of life, lack of personal space, etc. The instructions for the associative test did not contain any mention of urban environment or city. In addition, the presence of ambivalent elements in the core indicates that the social representation is not yet fully formed. All of these facts show that research of this phenomenon should be continued on both theoretical and empirical levels.

Understanding of “satiety” in the group of respondents is, firstly, stereotypical (young people interpret it literally—they give a large or even an excessive number of stimuli as manifestations of urban environment (social, spatial, informational environment); secondly, it is ambivalent in terms of emotional experience and, thirdly, it includes sources of social-psychological satiety with the living conditions.

### 3.1. Manifestations of Social-Psychological Satiety with the Living Conditions and Its Sources

Respondents describe all manifestations of social-psychological satiety with the living conditions as attitudes to behavioral responses. The most overt of them are: “Desire to go somewhere”, “try to communicate only with close friends and relatives”, “actively looking for new sources of pleasure”, and “constantly feeling of negative emotions, annoyed by everything around”. Less overt are such manifestations as “desire to change activity or situation” or “feeling bored”. The least overt is the readiness to avoid situations, which appeared as sources of satiety: “Willingness to shut off from everything and everyone” or “reluctance to see and hear anything and anyone”.

In summary, we can say that satiety mostly manifests as a desire to shut off from everything and everyone, change activities or situations, the wish to communicate only with close friends and relatives, and the search for new sources of pleasure.

### 3.2. Sources of Satiety with the Living Conditions

Most mentioned sources are monotonous living conditions, climatic conditions, and high rate of life. Results show that satiety is caused not only by an overflow of information, but also by forced everyday monotony of life (repeating schedule and uncomfortable weather conditions). It is important to mention that the described sources are specific for respondents’ age group. Young people are oriented towards a search of diversity in ways to structure free time, etc. The nature of the climate in the region, regularly subjected to weather manipulations on days when city events are held, also manifests as a source of satiety. Weather conditions, produced by lack of sunlight and bright colors, likely also correlate with the manifestation of satiety with the living conditions. A high rate of life, as one of attributes of the modern city, increases social-psychological satiety. Indeed, in spite of everyday monotony (home–work/university/home), everyone in the modern city is forced to hurry, because the distance between home and work is typically significant. In addition, the Moscow Metropolitan is one of the world’s most overcrowded municipal transport systems. The resulting physical and mental fatigue of modern citizens is compensated by a decrease in the severity of perception and emotional experience of what is happening. All of these lead to social-psychological satiety as a defensive response of the psyche. It is important to notice that crowding is one of the main sources of social-psychological satiety. This phenomenon is well-known in social psychology. However, whereas we know that animals are prone to crowding (for example, rats become very aggressive when they are crowded) [20], studies of crowding of people are not so univocal—crowding affects psychological comfort, behavior, and task performance, but we cannot say that this effect is completely negative [18,19,21,22,23,24].

### 3.3. Analysis of Correlations Between Sources and Manifestations of Social-Psychological Satiety

Correlation analysis between sources and manifestations allowed us to get the following results (Table 2). We can distinguish several forms of social-psychological satiety. So, social-psychological satiety as feeling negative emotions is caused by aggressive advertisement, close constant contacts with large numbers of people, monotony, and high rate of life. All of these phenomena are characteristics of urban environments. The states of apathy and boredom are caused by monotonous rate of life, climatic conditions, and forced contacts with people. Advertisement, monotony of life, and forced contacts with people cause reluctance to see and hear anything and anyone, and willingness to shut off from everything and everyone. So, an emotional form of social-psychological satiety can be interpreted as a precondition to the desire to retreat somewhere away from the city.

Behavioral manifestations of social-psychological satiety with the living conditions also vary. An active search for new sources of pleasure is caused by the many opportunities for free pastimes and goods, as well as different fashion tendencies. This can be exemplified by the many sensational and well-publicized media stories from the lives of rich kids that deliberately violate moral and legal norms of behavior in society. Willingness to change activities is caused by several phenomena—monotony of life, high rate of life, forced contacts with people, and the many opportunities for free pastimes and goods.

These results show that in general, social-psychological satiety with the living conditions in the modern city is caused by high rate of life, climatic conditions, and forced contacts with people. However, behavioral manifestations of social-psychological satiety (willingness to change activities and actively search for new sources of pleasure) are also caused by large amounts of goods, opportunities for free pastimes, and forced informatization of the urban environment.

## 4. Discussion

Summarizing the data analysis, it is important to mention that this work should be defined as exploratory research due to the insufficient development of the phenomenon. This study was devoted to solving the following tasks: The operationalization of satiety as a social-psychological phenomenon, the search for methodological tools to analyze satiety as a group phenomenon, and the development of an approach to the identification of forms of satiety.

The low number of respondents, specifically the sample of citizens aged 18 to 30, makes it hard to transfer results to the whole general population. The questionnaires that we used for this research were made on the basis of focus group conclusions, so we cannot be totally sure of the complete validity and reliability of these methods.

According to our results, satiety with the living conditions appears as a complex psychological state, which is caused by the severity of events in urban environments—forced communication, overflow of information, forced informatization, high rate of life, etc. In addition, the mention of social sources is found in social representations of young citizens—they were included in the peripheral zone, which, according to other researchers [16], performs the function of group adaptation to environmental changes. It was found that social-psychological satiety has different forms, both as emotional responses and as readiness for further behavioral reactions.

## 5. Conclusions

The correlations allowed us to formulate the following conclusions:

The structure of social representation clearly shows that social-psychological satiety is connected with urban environment—both the core and periphery include different assessments about different attributes of modern cities. In addition, structure includes ambivalent assessments about emotional manifestations of social-psychological satiety—it indicates that social representation is not yet fully formed.

The correlation analysis allowed us to draw the following conclusion—social-psychological satiety manifests as an emotional response and readiness for behavioral reactions. Emotional manifestations of satiety include aggression, irritation, and apathy produced by annoying advertisements in urban environments, monotonous conditions, a high rate of life, constant forced contact with large numbers of people, and climatic conditions. Satiety as readiness for behavioral responses can be divided into several forms: The active search for new sources of pleasure is caused by the huge numbers of alternatives (products, services, pastimes, etc.) and different fashion tendencies (music, films, clothes, books, etc.). The intention to leave the city for some time is caused by the monotonous conditions and high rate of life, constant forced contact with large numbers of people, and overflow of information and alternatives.

The general conclusion of this research is that socio-psychological satiety is a new, complex, and actually existing phenomenon. Satiety is closely connected with different attributes of modern cities; further study of this phenomenon can be very useful—it can shed light on the correlation between developing urban environments and the social and psychical well-being of citizens.

## Figures and Tables

**Table 1:** Structure and content of social representation of satiety among young Moscovites.

Average Rank			
**Frequency of References**		**<1.9**	**>1.9**
	>17.5	A sufficient amount, an excess of something Stress, depression, tiredness, joy, pleasure, satisfaction	Indifference, boredom, apathy, monotony, the desire to give up everything
	<17.5	People, crowd, excess of people Communication, relationship Concentration, cheerfulness Private space, personal home Wealth, money	Expressions of hostility, aggression Negative characteristics of people Positive thoughts, associations Internet, information, advertising Transport, the rhythm of life, urban environment and its characteristics Work, education

**Table 2:** Correlations between sources and manifestations of satiety (*p* < 0.05 *; *p* < 0.001 **).

	I don’t Want to See and Hear Anything	Ready to Close from Everything and Everyone	Actively Looking for Other Sources of Pleasure in the City	I Want to go Somewhere	Feeling Bored	I try to Communicate Only with Relatives and Friends	Resolutely Set to Change Activities	I Constantly Experience Negative Emotions, Everything Around Is Annoying
Advertising in the city	0.39 **	0.38 **	−0.32	0.43	0,166	0.25 *	0.177	0.31 *
Monotony of life	0.37 **	0.33 **	0.27 *	0.35 *	0.28 *	0.17	0.33 *	0.31 *
Forced daily contact with large numbers of people	0.19	0.31 *	0.116	0.095	0.23 *	0.093	0.25 *	0.27 *
Different fashion tendencies	0.018	0.1	0.24 *	0.08	0.002	0.1	0.19	0.15
Too many alternatives of choices (pastimes, products, etc.)	−0.062	0.13	0.30 *	0.06	0.04	−0.7	0.23 *	0.02
Overflow of information	0.05	0.18	0.16	0.12	0.12	0.02	0.20 *	0.16
Climatic conditions	0.005	0.11	0.19	0.17	0.22 *	0.07	0.05	−0.07
High rate of life	0.21	0.29 **	0.09	0.14	0.14	0.005	0.29 *	0.27 *

## Data Availability

The datasets used and analyzed during the current study are available from the corresponding author on reasonable request.
